# Development of a prognostic index based on an immunogenomic landscape analysis of papillary thyroid cancer

**DOI:** 10.18632/aging.101754

**Published:** 2019-01-20

**Authors:** Peng Lin, Yi-nan Guo, Lin Shi, Xiao-jiao Li, Hong Yang, Yun He, Qing Li, Yi-wu Dang, Kang-lai Wei, Gang Chen

**Affiliations:** 1 Department of Medical Ultrasonics, First Affiliated Hospital of Guangxi Medical University, Nanning, Guangxi Zhuang Autonomous Region 530021, PR China; 2 Department of Pathology, First Affiliated Hospital of Guangxi Medical University, Nanning, Guangxi Zhuang Autonomous Region 530021, PR China; 3 Department of Pathology, Second Affiliated Hospital of Guangxi Medical University, Nanning, Guangxi Zhuang Autonomous Region 530021, PR China; 4 Department of PET/CT, First Affiliated Hospital of Guangxi Medical University, Nanning, Guangxi Zhuang Autonomous Region 530021, PR China

**Keywords:** papillary thyroid cancer, immunogenomic landscape, The Cancer Genome Atlas, prognostic index, personalized medicine

## Abstract

Background: Papillary thyroid cancer (PTC) is the most common subtype of thyroid cancer, and inflammation relates significantly to its initiation and prognosis. Systematic exploration of the immunogenomic landscape therein to assist in PTC prognosis is therefore urgent. The Cancer Genome Atlas (TCGA) project provides a large number of genetic PTC samples that enable a comprehensive and reliable immunogenomic study.

Methods: We integrated the expression profiles of immune-related genes (IRGs) and progression-free intervals (PFIs) in survival in 493 PTC patients based on the TCGA dataset. Differentially-expressed and survival-associated IRGs in PTC patients were estimated a computational difference algorithm and COX regression analysis. The potential molecular mechanisms and properties of these PTC-specific IRGs were also explored with the help of computational biology. A new prognostic index based on immune-related genes was developed by using multivariable COX analysis.

Results: A total of 46 differentially expressed immune-related genes were significantly correlated with clinical outcome of PTC patients. Functional enrichment analysis revealed that these genes were actively involved in a cytokine-cytokine receptor interaction KEGG pathway. A prognostic signature based on RGs (AGTR1, CTGF, FAM3B, IL11, IL17C, PTH2R and SPAG11A) performed moderately in prognostic predictions and correlated with age, tumor stage, metastasis, number of lesions, and tumor burden. Intriguingly, the prognostic index based on IRGs reflected infiltration by several types of immune cells.

Conclusions: Together, our results screened several IRGs of clinical significance, revealed drivers of the immune repertoire, and demonstrated the importance of a personalized, IRG-based immune signature in the recognition, surveillance, and prognosis of PTC.

## Introduction

Thyroid cancer accounts for > 90% of endocrine system malignancies, and its incidence rate is on the rise [1–5]. In the United States, it is estimated that 53,990 new cases will be diagnosed and 2,060 thyroid cancer fatalities will occur in 2018 [6]. Papillary thyroid carcinoma (PTC), the most common subtype of thyroid cancer, accounts for 80% of reported cases [7–11]. At present, patients with PTC typically undergo surgical treatment and radioactive iodine therapy, with an excellent overall prognosis [12,13]. Although the majority of PTCs remain indolent, tumor recurrence and metastasis stymies clinical management and survival in certain patients [14–17]. Existing treatments are insufficient for patients with locally advanced or distantly metastatic PTC. Careful monitoring of the progression of PTC with the help of novel and sensitive biomarkers could reduce numbers of PTC patients not diagnosed before the onset of aggressive disease.

Cancer immunotherapy has been a major driver of personalized medicine, with aggressive efforts to leverage the immune system to fight tumors [18,19]. In recent decades, immunotherapy developments have entered the treatment protocols of many types of cancers [20,21]. At present, cutting-edge immunotherapies promise PTC patients another alternative treatment method. In vitro and/or in vivo experiments proposed that immune checkpoint inhibitors could boost the effective elimination of thyroid tumor cells [22,23]. Besides, Bai Y et al. found that the expression of BRAF V600E is positively correlated with PD-L1/PD-1 in PTC samples, which indicated that immune checkpoint therapy might be effective for PTC patients with the BRAF V600E mutation [24]. More importantly, several ongoing clinical trials have fueled the field of tumor immunology in thyroid cancer [25]. The thyroid gland is the largest endocrine organ in the human body, and is a frequent target of autoimmune disease. Immunological cells are widespread in the thyroid cancer microenvironment [26]. Certain studies have proposed that chronic lymphocytic thyroiditis, a common autoimmune disease, may trigger or accelerate development of PTC [27,28], although a potential causative relationship therein has yet to be established. While these findings support the importance of immunology in PTC, molecular mechanisms still remain unclear, particularly for with regards to immunogenomic effects. With the advent of public, large-scale gene expression datasets, cancer researchers have been able to identify culpable biomarkers for tumor monitoring and surveillance with great speed and accuracy [29,30]. Li et al. (2017) comprehensively explored the prognostic value of immune-related genes (IRGs) to develop an individualized immune signature which could improve prognostic estimations for patients with nonsquamous non–small cell lung cancer [31]. However, the clinical relevance and prognostic significance of IRGs in PTC has yet to be explored.

Our aim in this investigation is to gain insight into the potential clinical utility of IRGs on prognostic stratification and their implicational potential as biomarkers for targeted PTC therapy. We integrated IRG expression profiles with clinical information, applying computational methods for the assessment of progression-free intervals (PFIs) in PTC patients. We systematically analyzed the expression status and prognostic landscape of IRGs and developed an individualized prognostic signature for PTC patients. Bioinformatics analyses were conducted to explore underlying regulatory mechanisms. Results from this study could offer a foundation for subsequent, in-depth immune-related work with great promise for personalized medicine in the treatment of PTC.

## RESULTS

### Identification of differentially expressed IRGs

**Figure 1:**
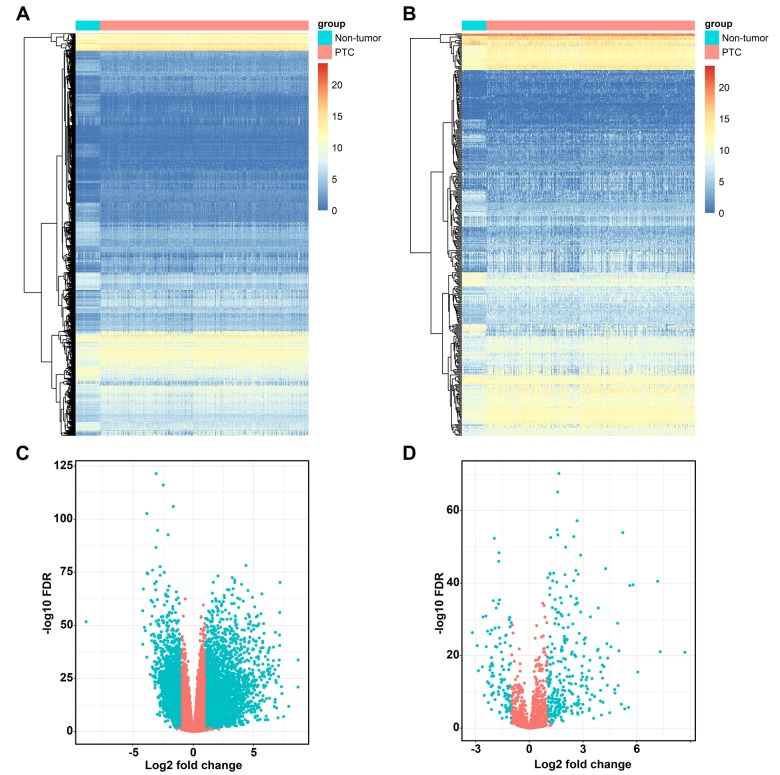
**Differentially expressed immune-related genes.** Heatmap (**A**) and volcano plot (**C**) demonstrating differentially expressed genes between papillary thyroid cancer (PTC) and non-tumor tissues, blue dots represent differentially expressed genes and red dots represent no differentially expressed genes. Differentially expressed immune-related genes (IRGs) are shown in heatmap (**B**) and volcano plot (**D**), blue dots represent differentially expressed genes and red dots represent no differentially expressed genes.

**Figure 2:**
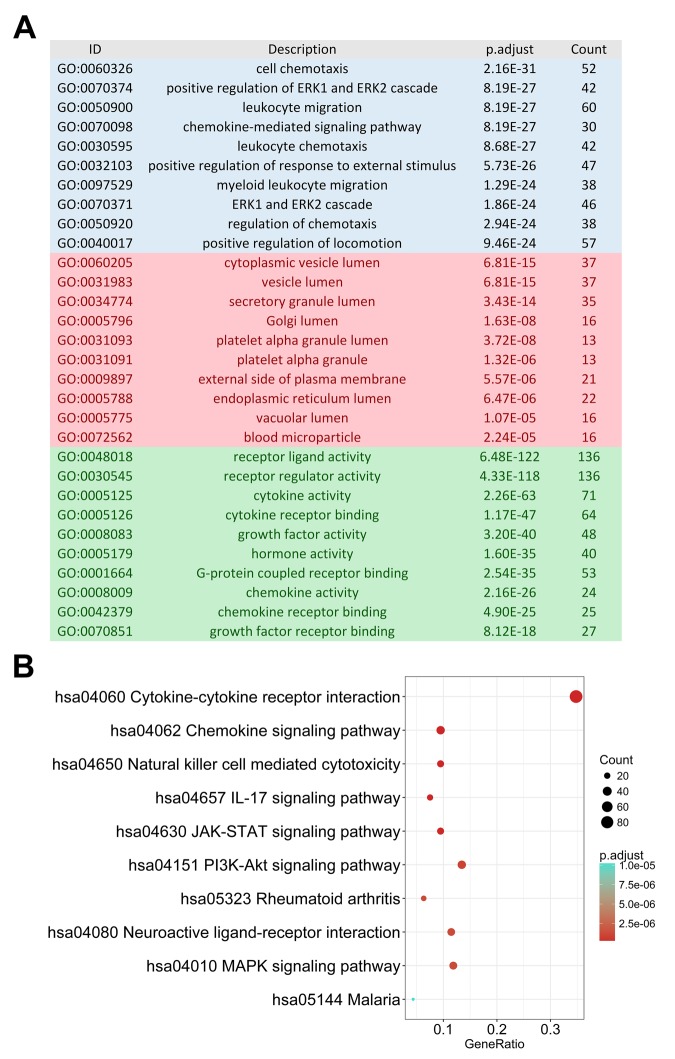
**Gene functional enrichment of differentially expressed immune-related genes.** (**A**) Gene ontology analysis; blue, red and green bars represent biological process, cellular component and molecular function, respectively. (**B**) The top 10 most significant Kyoto Encyclopedia of Genes and Genomes pathways.

The edgeR algorithm identified 5,498 differentially expressed genes, 2,258 up-regulated and 3,240 down-regulated (Figures 1A and 1C). From this set of genes, we extracted 374 differentially expressed IRGs, including 269 up-regulated and 105 down-regulated (Figures 1B and 1D). As expected, gene functional enrichment analysis revealed that inflammatory pathways were most frequently implicated. “Cell chemotaxis,” “cytoplasmic vesicle lumen,” and “receptor ligand activity” were the most frequent biological terms among biological processes, cellular components, and molecular functions, respectively (Figure 2A). For the Kyoto Encyclopedia of Genes and Genomes (KEGG) pathways, cytokine-cytokine receptor interactions were most often enriched by differentially expressed IRGs (Figure 2B).

### Identification of survival-associated IRGs

**Figure 3:**
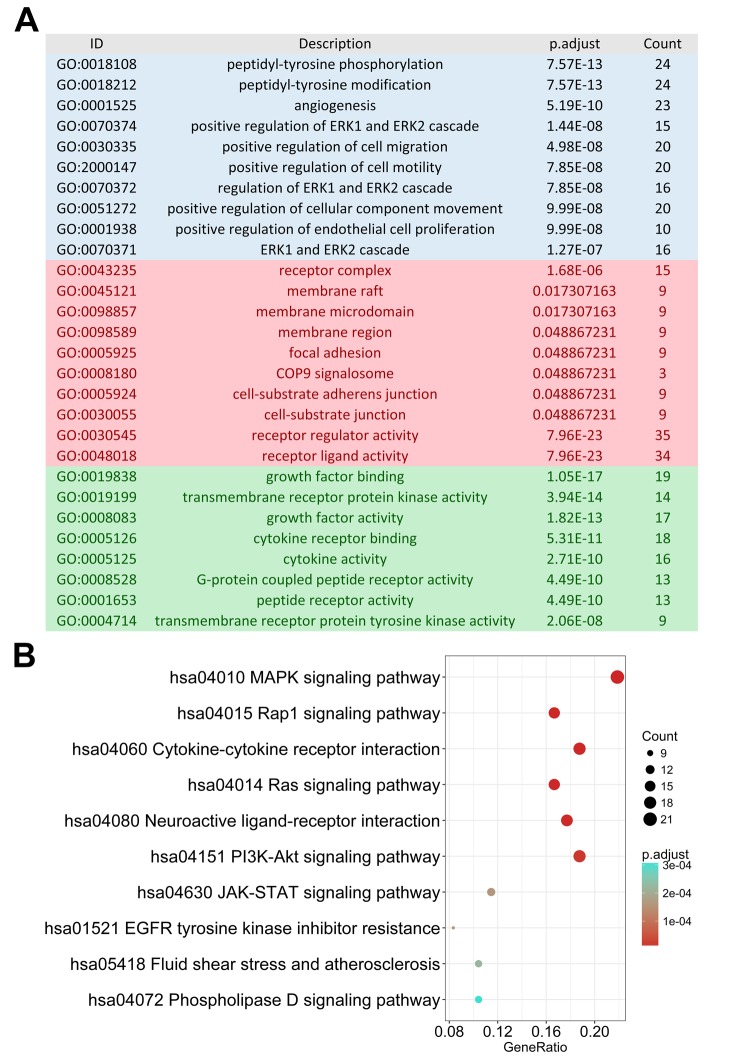
**Gene functional enrichment of survival-associated immune-related genes.** (**A**) Gene ontology analysis; blue, red and green bars represent biological process, cellular component and molecular function, respectively. (**B**) The top 10 most significant Kyoto Encyclopedia of Genes and Genomes pathways.

As monitoring disease outcome is important for clinical management, we focused our efforts on uncovering molecular biomarkers that could serve as viable prognostic indicators. After screening, we found 130 IRGs that were significantly correlated to PFI in PTC patients. Similar to the results from our gene enrichment analysis of differentially expressed genes, we found these survival-associated IRGs was most enriched in several gene ontology (GO) terms related to cell interaction and movement. The MAPK signaling pathway was the most frequently identified KEGG pathway (Figure 3).

### Identification of hub IRGs

**Figure 4:**
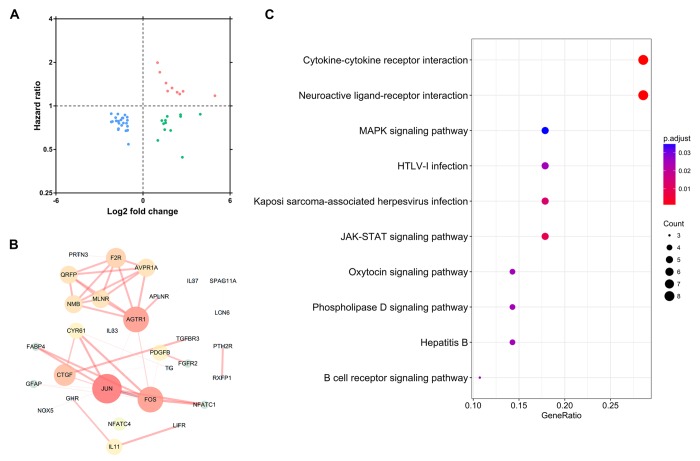
**Identification of hub immune-related genes.** (**A**) The intersection of differentially expressed IRGs and survival-associated IRGs. (**B**) Protein-protein interaction network of hub IRGs. (**C**) Kyoto Encyclopedia of Genes and Genomes pathway analysis of hub IRGs.

To extract IRGs which actively participated in the onset and progression of PTC, we chose differentially expressed IRGs which were significantly correlated with clinical outcomes (P<0.05). A total of 46 IRGs met this criterion (Table 1; Figure 4A). Protein-protein interaction (PPI) network analysis demonstrated that JUN, AGTR1, and FOS were the three hub genes among these this dataset (Figure 4B). Functional enrichment analysis revealed that these genes were actively involved in a cytokine-cytokine receptor interaction KEGG pathway (Figure 4C).

**Table 1:** General characteristics of PTC-specific immune-related genes.

Gene symbol	logFC	FDR	HR	Z-value	P-value
ACKR2	-1.687064217	4.26E-49	0.696192037	-2.36286581	0.018134235
GHR	-1.70049177	9.79E-47	0.685921851	-2.52786588	0.011475818
PLXNA3	1.160096051	2.46E-39	1.710549504	1.982939323	0.047374208
JUN	-1.655315234	4.09E-36	0.758345613	-2.052977898	0.040074721
ANGPTL1	-1.713306014	5.04E-35	0.783338142	-2.687442591	0.007200148
LIFR	-1.841039853	7.26E-34	0.788836186	-2.087231692	0.036867196
QRFP	-1.088374171	2.47E-31	0.676925655	-2.007271602	0.044720751
F2R	1.49644826	5.58E-31	0.685775226	-2.420224391	0.015510932
TGFBR3	-1.00520471	7.94E-30	0.5433445	-3.375986425	0.000735515
FGFR2	-1.068993458	1.13E-28	0.72401054	-2.004848809	0.044979213
CYR61	-1.685846521	1.78E-28	0.785164478	-2.124743187	0.033608048
LTF	-2.184099931	1.05E-26	0.775433507	-2.735194157	0.006234349
ESRRG	-1.34743252	2.5E-24	0.762353203	-2.941532873	0.003265922
NR1D1	2.002775191	3.32E-24	1.330937381	2.348481695	0.018850126
NFATC1	-1.053766381	1.97E-23	0.67892188	-2.367286938	0.017919034
AVPR1A	-1.880477808	3.14E-23	0.829758857	-2.493237563	0.012658412
PDGFB	1.024220384	4.43E-23	0.577751758	-2.495217245	0.012588004
APLNR	1.419410262	1.07E-22	0.753698008	-2.259657038	0.023842545
CRABP2	2.356586586	4.05E-21	1.243529964	2.379146595	0.017352773
PRTN3	2.534878013	6.08E-21	1.209363644	2.026378898	0.042725972
ULBP1	2.594323933	1.83E-20	0.84592328	-2.100203699	0.035710926
MLNR	-2.104936046	3.53E-20	0.780100381	-2.269458162	0.023240479
LCN6	3.952072642	3.84E-20	0.8764868	-2.249166036	0.024501934
CTGF	-1.469118649	1.13E-19	0.734379979	-3.093995395	0.001974806
NFATC4	1.004724232	2.32E-18	1.992123979	3.548274944	0.000387763
RXFP1	-1.517269947	8.68E-18	0.800928632	-2.659068477	0.007835704
IL33	-1.137266176	7.3E-17	0.758980347	-2.282888161	0.022436957
FOS	-1.427430135	7.51E-17	0.825967325	-2.361681281	0.018192275
GDF10	-2.185523784	9.59E-17	0.883881161	-2.091835846	0.036453203
FAM3B	-1.381253974	3.69E-14	0.825630975	-2.379265083	0.017347196
NOX5	2.588531962	5.89E-13	0.869857077	-2.174717678	0.029651279
IL37	4.958949015	1.81E-12	1.176018299	2.90407439	0.003683406
TG	-1.082464079	2.11E-12	0.835915361	-2.360188443	0.018265654
CMTM5	-1.176756666	1.16E-11	0.673526685	-2.951366659	0.003163711
CRABP1	-1.727556563	8E-11	0.878968593	-2.738050559	0.006180457
IL17C	1.586653222	1.57E-10	1.44017285	3.26184305	0.001106904
GFAP	1.549182091	9.98E-10	0.795114413	-2.060125029	0.03938659
NMB	1.295911066	4.07E-09	0.791911007	-2.503776648	0.012287558
IL11	1.699033239	0.000000013	1.266514138	2.902922992	0.003696975
IL36A	2.7678365	1.81E-08	1.261659287	2.448034461	0.014363792
SCGB3A1	1.673414287	0.000000171	0.846990277	-2.322810119	0.020189355
PTH2R	1.563089171	0.00000164	0.76502175	-2.128669251	0.033281635
FABP4	-1.239736536	0.00000426	0.865867291	-2.745652317	0.006039073
AGTR1	-1.076480549	0.0000109	0.795822713	-3.63116516	0.000282145
LCN15	1.907212684	0.0000225	0.677152854	-2.340634808	0.019250987
SPAG11A	2.715397978	0.00014475	0.441582959	-2.183332337	0.029011345

### Characteristics of hub IRGs

**Figure 5:**
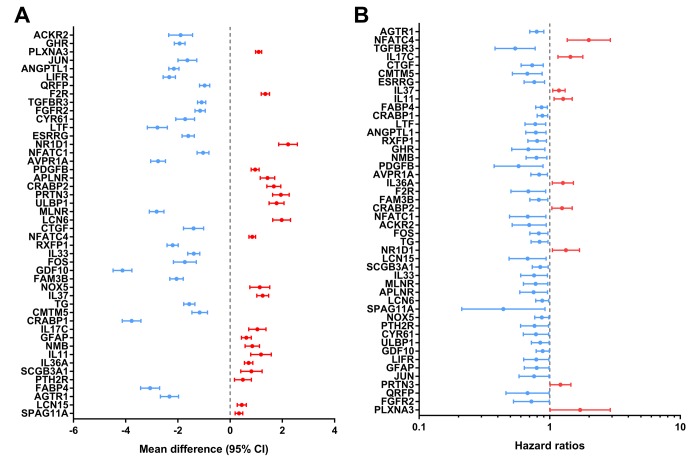
**Expression profiles and prognostic values of hub immune-related genes.** (**A**) Forest plot of mean difference showing gene differences between PTC and non-tumor samples. (**B**) Forest plot of hazard ratios showing the prognostic values of genes.

**Figure 6:**
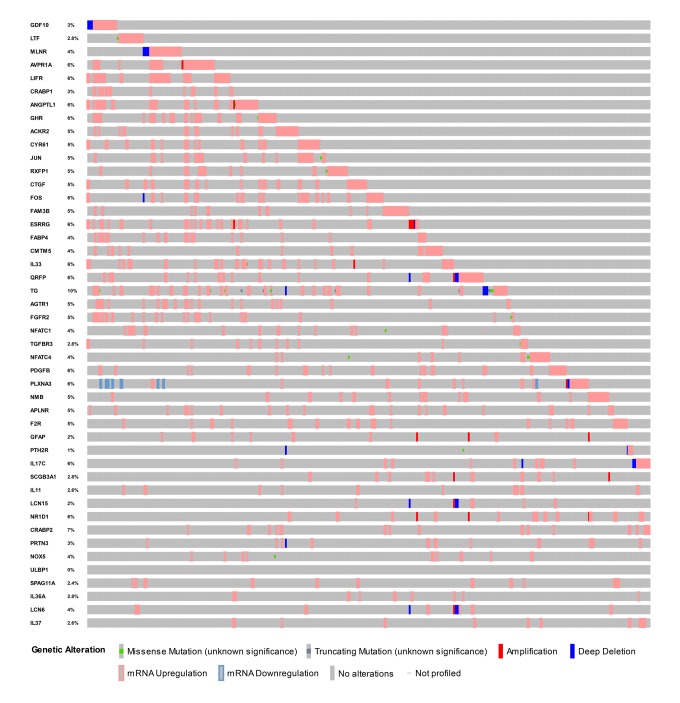
**Mutation landscape of hub immune-related genes.** TG is the gene with the highest mutation frequency. And there were 25 genes with a mutation rate ≥ 5%.

Identified hub IRGs possess excellent biomarker potential for monitoring prognosis. A forest plot of expression profiles revealed that most of the identified hub IRGs were up-regulated in PTC samples (Figure 5A). A forest plot of hazard ratios indicated that most of these genes were protective factors (Figure 5B). Owing to the significant clinical value of these IRGs, we embarked on a comprehensive exploration of their molecular characteristics. We examined genetic alterations of these genes and found that mRNA upregulation and deep deletion were the two most commonly occurring types of mutation (Figure 6).

### TF regulatory network

**Figure 7:**
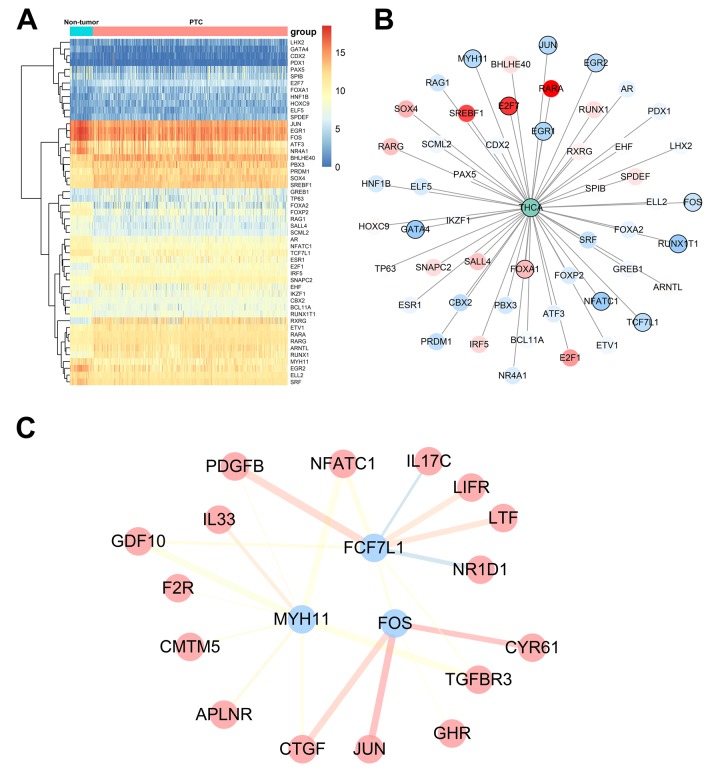
**Transcription factor-mediated regulatory network.** (**A**) Differentially expressed transcription factors (TFs). (**B**) Survival analysis of differentially expressed TFs. (**C**) Regulatory network constructed based on clinically relevant TFs and IRGs.

To explore potential molecular mechanisms corresponding to the clinical significance of our hub IRGs, we investigated the regulatory mechanisms of these genes. We examined the expression profiles of 318 transcription factors (TFs) and found that 51 were differentially expressed between PTC and non-tumor thyroid samples (Figure 7A). Among these 51 TFs, 11 were correlated to the PFI of PTC patients (Figure 7B). We then constructed a regulatory network based on these 11 TFs and our 46 hub IRGs. A correlation score more than 0.4 and combined score more than 0.6 were set as the cut-off values. The TF-based regulatory schematic acutely illustrated the regulatory relationships among these IRGs (Figure 7C).

### Evaluation of clinical outcomes

**Figure 8:**
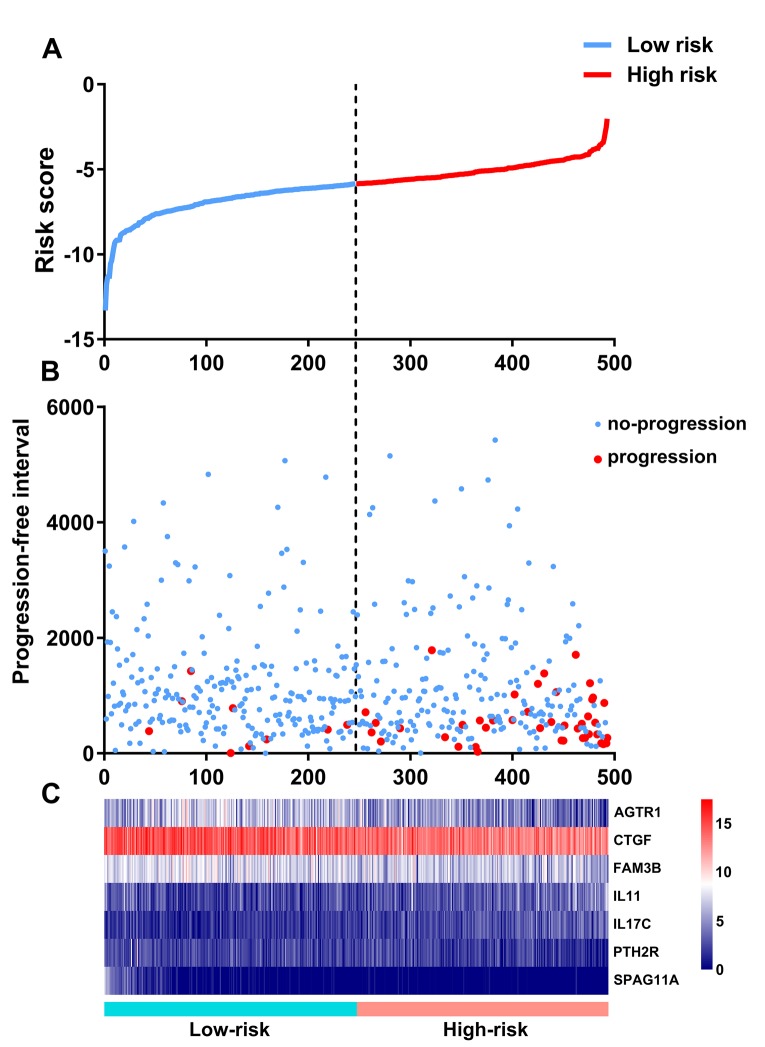
**Development of the prognostic index based on immune-related genes.** (**A**) Rank of prognostic index and distribution of groups. (**B**) Survival status of patients in different groups. (**C**) Heatmap of expression profiles of included genes.

Based on the results of multivariate Cox regression analysis, we constructed a prognostic signature to separate the PTC patients into two groups with discrete clinical outcomes with regards to PFI (Figure 8). The formula was as follows:

[Expression level of AGTR1 * (-0.1212)] + [Expression level of CTGF * (-0.3284)] + [Expression level of FAM3B * (-0.1675)] + [Expression level of IL11 * 0.3089] + [Expression level of IL17C * 0.2368] + [Expression level of PTH2R * (-0.2823) + [Expression level of SPAG11A * (-0.9550)]

**Figure 9:**
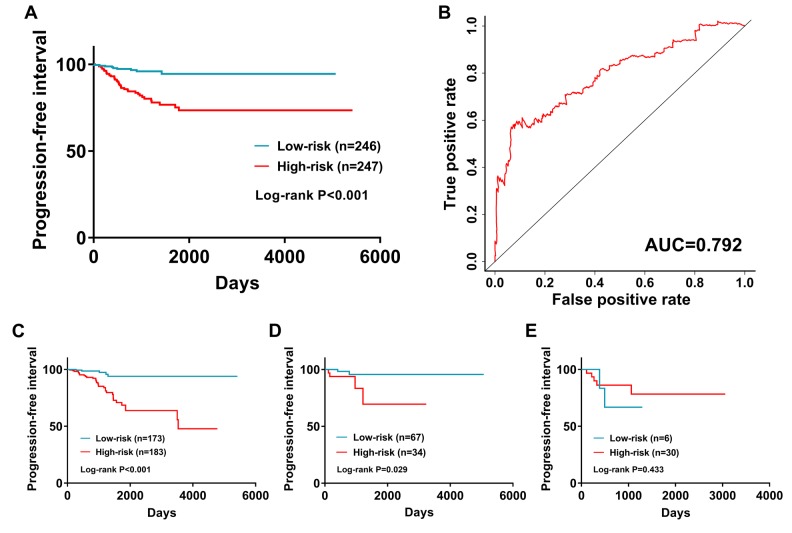
**The prognostic value of prognostic index.** (**A**) Patients in high-risk group suffered shorter progression-free intervals. (**B**) Survival-dependent receiver operating characteristic (ROC) curve validation of prognostic value of the prognostic index. Subgroup analysis performed in (**C**) Classical; (**D**) Follicular; and (**E**) follicular subtypes of PTC.

This immune-based prognostic index could be an important tool for distinguishing among PTC patients based on potential discrete clinical outcomes (Figure 9A). The area under curve of the receiver operating characteristic (ROC) curve was 0.792, suggesting moderate potential for the prognostic signature based on IRGs in survival monitoring (Figure 9B). Multivariate Cox regression analysis suggested that the prognostic signature could become an independent predictor after other parameters were adjusted, including age, gender, pathologic stage, tumor stage, lymph node metastasis status, distant metastasis status, tumor size, tumor status and the amounts of nodules (Table 2). The prognostic signature was also found to be a moderately viable index for different PTC subtype (classical, follicular, and tall cell) patients (Figure 9C - E). We also explored the clinical significance of included genes (Table3).

**Table2:** Univariate and multiple regression analysis of papillary thyroid carcinoma.

Variables	Univariate analysis		Multivariate analysis	
	Hazard ratio (95% CI)	*P* value	Hazard ratio (95% CI)	*P* value
Age	1.019 (1.002-1.036)	0.029	1.003 (0.958-1.050)	0.901
Gender (male/female)	1.635 (0.920-2.906)	0.094	1.972 (0.773-5.036)	0.156
Pathologic stage	1.578 (1.249-1.994)	<0.001	1.272 (0.654-2.471)	0.478
Tumor stage	1.989 (1.443-2.742)	<0.001	0.892 (0.476-1.670)	0.721
Lymph node metastasis (yes/no)	1.679 (0.932-3.024)	0.084	1.342 (0.504-3.575)	0.556
Distant metastasis (yes/no)	7.559 (2.857-20.004)	<0.001	1.085 (0.193-6.110)	0.926
Tumor size	1.182 (0.997-1.401)	0.060	0.573 (0.371-.886)	0.012
Tumor status (with tumor/tumor free)	16.169 (9.243-28.285)	<0.001	15.152 (4.765-48.173)	<0.001
Multifocality (multifocal/unifocal)	1.095 (0.627-1.913)	0.750	1.176 (0.435-3.174)	0.750
IRGPI	2.711 (2.046-3.592)	<0.001	2.425 (1.492-3.943)	<0.001

**Table 3:** Relationships between the expressions of the immune-related genes and the clinicopathological factors in papillary thyroid cancer.

Genes	Age (≥60/ <60)	Gender (male/ female)	Tumor status (with tumor/ tumor free)	Primary neoplasm focus type (multifocal/ unifocal)	Pathological stage (IV–III/ I–II)	T stage (T3–T4/ T1–T2)	N stage (N1–3/ N0)	M stage (M1/ M0)

t	P	t	P	t	P	t	P	t	P	t	P	t	P	t	P
AGTR1	-0.422	0.673	-2.103	**0.036**	-2.145	**0.032**	3.444	**0.001**	-3.615	<0.001	-4.832	**<0.001**	-5.088	**<0.001**	-3.861	**<0.001**
CTGF	-0.513	0.608	-0.895	0.371	-0.105	0.916	1.010	0.313	-0.405	0.686	-0.853	0.394	1.288	0.198	-1.237	0.217
FAM3B	-0.214	0.831	-1.261	0.208	-2.667	**0.008**	0.547	0.584	0.272	0.786	0.225	0.822	0.987	0.324	-1.589	0.113
IL11	1.340	0.182	1.377	0.169	3.212	**0.002**	-0.510	0.610	3.652	**<0.001**	3.580	**<0.001**	5.146	**<0.001**	1.880	0.096
IL17C	0.878	0.380	1.260	0.208	2.144	**0.037**	-2.431	**0.015**	3.746	**<0.001**	3.918	**<0.001**	4.199	**<0.001**	0.899	0.370
PTH2R	-2.093	**0.037**	0.481	0.631	-2.729	**0.007**	-0.125	0.901	-1.480	0.140	0.121	0.904	1.968	0.050	-0.538	0.591
SPAG11A	-2.551	**0.013**	1.884	0.061	-0.198	0.843	1.025	0.306	-1.587	0.113	-0.586	0.558	0.756	**0.450**	-0.153	0.879

Note: t: t value of student's t test; P: P-value of student's t test.

### Clinical utility of prognostic signature

**Figure 10:**
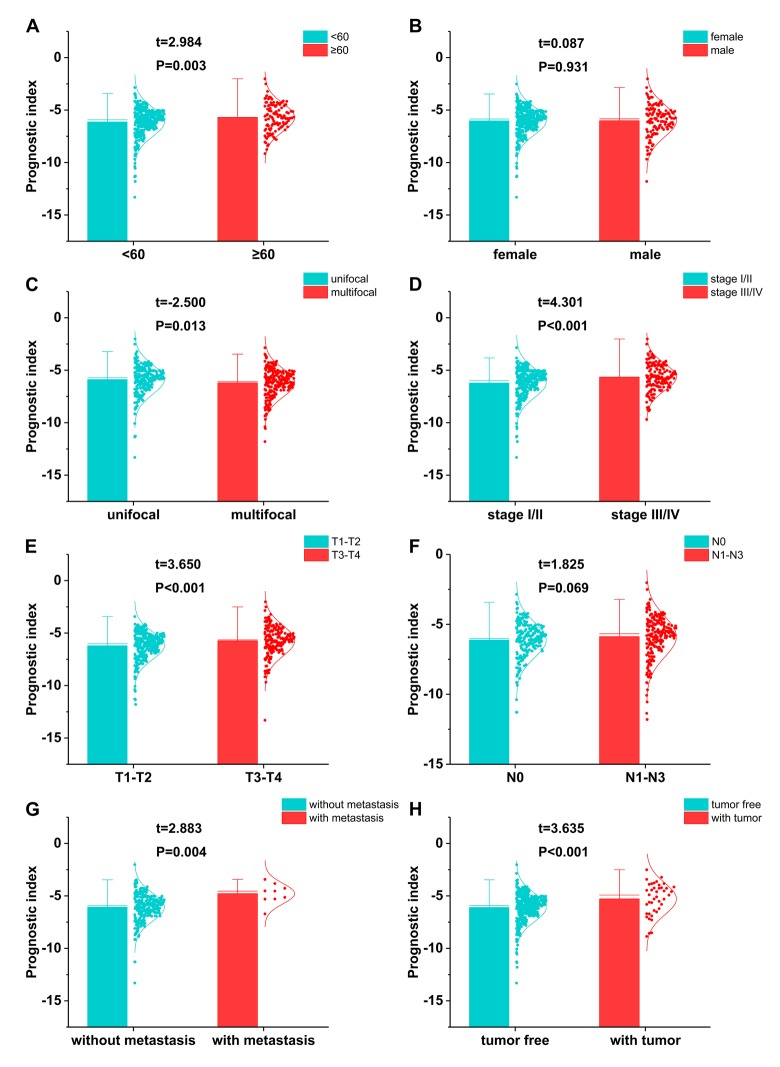
**The relationships between the immune-based prognostic index and** (**A**) age; (**B**) gender; (**C**) number of lesions; (**D**) tumor stage; (**E**) T stage; (**F**) lymph node metastasis; (**G**) distant metastasis; and (**H**) tumor burden.

**Figure 11:**
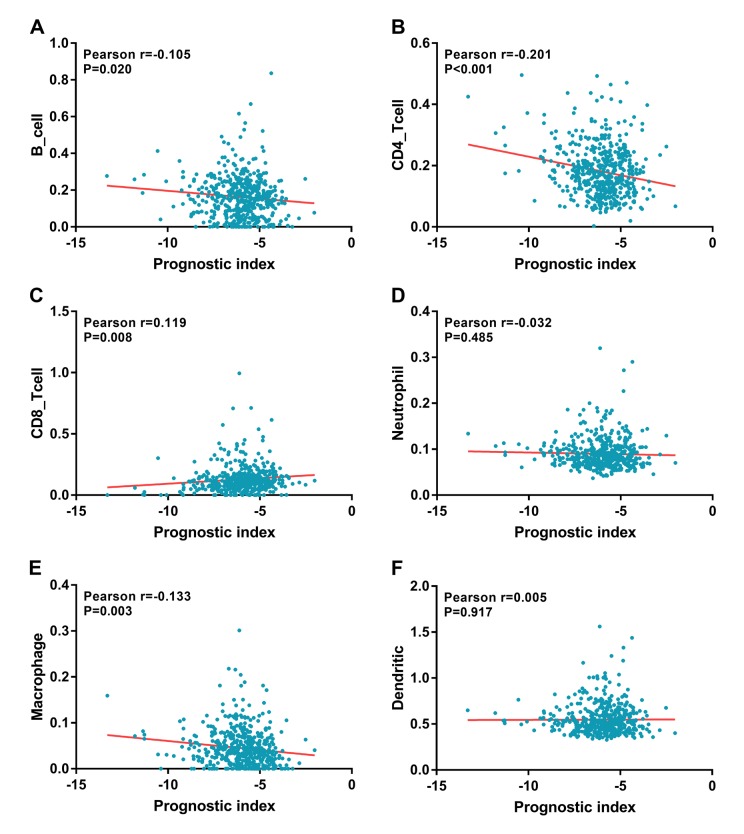
**Relationships between the immune-related prognostic index and infiltration abundances of six types of immune cells. The correlation was performed by using Pearson correlation analysis.** (**A**) B cells; (**B**) CD4 T cells; (**C**) CD8 T cells; (**D**) neutrophils; (**E**) macrophages; and (**F**) dendritic cells.

Relationships were analyzed between the immune-related gene-based prognostic index (IRGPI) and clinical and demographic characteristics, including age, gender, number of lesions, American Joint Committee on Cancer (AJCC) stage, and tumor burden. IRGPI was significantly higher in seniors (Figure 10A), multifocal patients (Figure 10C), advanced stage cases (Figure 10D), advanced T stage cases (Figure 10E), distant metastasis cases (Figure 10G), and increased tumor burden (Figure 10H). However, no difference was observed between genders (Figure 10B) or with regards to lymph node metastasis (Figure 10F). To see if the immunogenome accurately reflected the status of tumor immune microenvironment, we analyzed relationships between IRGPI and immune cell infiltration (Figure 11).

## DISCUSSION

Although the significance of IRGs in tumor progression and immunotherapeutics has been well-established, a comprehensive, genome-wide profiling study exploring their clinical significance and molecular mechanisms has not been conducted. This comprehensive, integrated analysis of IRGs in PTC enhances our understanding of their clinical significance and illuminates potential molecular characteristics. The large number of PTC samples we had access to for this study facilitated robust results. Determination of more precise PFI clinical endpoints helped to assess potential clinical outcomes in THCA patients, and these PFIs enabled the analysis of more endpoint events than simply overall survival. We exposed several IRGs significantly involved in the initiation and progression of PTC; these IRGs may serve as valuable clinical biomarkers. Bioinformatic systems will enable a more in-depth exploration of their molecular mechanisms. Most important, an individualized immune prognostic signature based on selected, differentially expressed IRGs was proposed to measure immune cells infiltration and assess potential clinical outcomes.

Since the start of the “War on Cancer,” our understanding of tumorigenesis and techniques for clinical management have progressed impressively, but many aspects of PTC immune-related molecular mechanisms remain unclear. The initiation of cancer cells often occurs in densely infiltrated inflammatory environments [32], which were the first clues that pointed our research toward differentially expressed IRGs. Other studies have uncovered differentially expressed genes between PTC and non-tumor samples [29,33], providing a fundamental understanding of the pathogenesis of PTC at the genetic level. However, the characteristics of IRGs in PTC had not been systematically explored up to this point. Tumor immune escape is an indispensable step in cancer progression. Recently, Na et al. (2018) proposed an ImmuneScore based on immune cell abundances as a means to characterize the immune microenvironment of PTC [34]. They proposed ImmuneScore based on immune cell intensity and explored the relationships between ImmuneScore and thyroid differentiation and BRAFV600E status. However, the present study mainly focused on the immunogenomic profiles and the corresponding clinical significance. The two studies explored the immune landscape of PTC from two distinct perspectives. Combination the two results could describe the immune status of PTC more comprehensively. Indeed, study on the tumor immune microenvironment is an important buttress to investigations into immunotherapeutic PTC management.

Acquisition of invasive traits in cancer cells depends upon a succession of alterations to the genome. We focused our investigation on alterations to immunogenomic profiles to uncover relationships between these profiles and the immune microenvironment, and to suggest potential clinical implications. Gene functional enrichment analysis suggested that these genes are mainly involved in cytokine-cytokine receptor interactions and chemokine signaling pathways.

Chemokines and their receptors actively participate in the pathogenesis of early-stage PTC; these are virtually always found to be altered in cancerous cells. Notably, these are correlated to invasion, aggression, and metastasis of PTC [14,35,36]. As such, these chemokines could also act as clinical biomarkers for monitoring metastasis, assessing survival, and uncovering potential drug targets [37,38]. Computational biological algorithms provided several clues suggesting that alterations to the immunogenome could promote the initiation of PTC via several inflammatory pathways.

Owing to the favorable prognosis of most PTC cases, studies focused on the selection of effective prognostic biomarkers is limited. However, cases of metastasis and recurrence continue to stymie current clinical management protocols. Considering the favorable clinical outcome, PTC patients did need a longer follow-up time to capture more OS events. In TCGA database, the numbers of OS and DFS events are small, Hence, PFI was the most suitable clinical endpoints for the current study. We selected PFI as the key clinical endpoint for observation of survival. Of the pathways implicated by IRGs, the MAPK signaling pathway was the most significantly correlated with survival-associated IRGs. The MAPK pathway is a conserved signal-transduction pathway. To date, thyroid carcinoma has been considered to be a predominantly a MAPK driven cancer, with approximately 70% of thyroid carcinomas associated with mutations that activate this pathway [39]. We explored the expression profiles, prognostic value, and mutational status thereof and uncovered valuable data ripe for future clinical exploration.

To explore underlying molecular mechanisms corresponding to potential clinical value, we constructed a TF-mediated network to expose vital TFs that could regulate identified hub IRGs. MYH11, FOS, and FCF7L1 featured prominently in this network. The ChIP-seq and co-expression-based TF-IRG regulatory networks we constructed will also help to inform and direct future mechanism analysis. Previously, a handful of immunological reports have suggested a possible connection between MYH11 and FCF7L1 and PTC, but associations with the FOS and MAPK pathway are new to immunological study of PTC [40]. Considering the potential molecular mechanism of the seven IRGs, no reports of the function and mechanism of AGTR1, FAM3B, PTH2R or SPAG11A have been published in PTC. However, among these seven IGRs, three of them have been studied, including CTGF, IL11 and IL17C. CTGF is upregulated in PTC and promotes the growth of PTC cells [41]. IL11 has been reported to play an oncogenic role in anaplastic thyroid carcinoma [42]. Moreover, IL17C overexpression was observed in differentiated thyroid cancer and associated with the recurrence and mortality [43]. Hence, previous studies provided limited information about the mechanisms of seven IRGs in PTC patients survival. In the functional enrichment analysis, MAPK pathway was the most significant pathway, we hypothesize that MAPK pathway may play an important role in the process.

To develop a simple and convenient protocol for monitoring the immune status and suggesting clinical outcomes in PTC patients, we created an immune-based prognostic signature. Previously, Bisarro et al. (2017) explored genome-wide DNA methylation profiling and proposed an algorithm to predict the recurrence of well-differentiated thyroid carcinoma [44]. Ab Mutalib et al. (2016) integrated microRNA, gene expression, and TF signatures to study the molecular mechanisms of PTC in patients with lymph node metastasis [45]. Cheng et al combined genomic alterations and clinical parameters to develop a risk index model that could monitor the progression of PTC [46]. Beyond that, several researchers also have proposed prognostic signatures for PTC patients’ survival prediction [47–49]. Comparing with previous publications, the present study proposed a signature that chose the PFI as the endpoint, which was the most suitable for PTC patients’ survival monitoring. Furthermore, the IRGPI could not only as a prognostic indicator, but also as an immune status indicator.

Our prognostic index, based on seven IRGs differentially expressed in PTC, demonstrated favorable clinical viability. Of interest, our data showed that IRGPI performed moderately in prognostic predictions and correlated with age, tumor stage, metastasis, number of lesions, and tumor burden. This tool could enable relatively rapid adjustments to treatment plans based on the immune cells infiltration levels reflected by the IRGPI. It has been well established that CD4^+^ T lymphocytes are able to recognize cancer antigens, and that activated M1-macrophages have displayed anti-tumor functions [50–52]. Our analysis indicated that the IRGPI was significantly negatively correlated to the infiltration of CD4^+^ T cells and macrophages. Characterization of the immune infiltration landscape is necessary for the investigation of tumor–immune interactions. We explored the relationships between IRGPI and immune cell infiltration to reflect the status of immune microenvironment of PTC. Interestingly, B cell, CD4 T cell and macrophage cell infiltration levels were significantly negatively correlated with IRGPI, while the infiltration level of CD8 T cells was evidently positively correlated with IRGPI. These results indicated that the lower infiltration levels of B cell, CD4 T cell, macrophage and higher CD8+ T cell might be observed in high-risk patients. Our results confirmed and expanded the findings of immune cells is being essential for PTC progression. These current results also suggested that the IRGPI owned the potential to act as predictor for immune cells infiltration elevation, which is line with previous reports. The role of immune cells in PTC has not be fully explored. Previously, Ehlers et al. demonstrated that the frequencies of TPO- and Tg-specific CD8+ T cells in PTC patients were largely increased compared to the healthy controls [53]. Aghajani MJ et al as they reported that patients with low CD8+ and CD3+ expression presented with a significantly higher incidence of lymph node metastasis and extrathyroidal extension in papillary thyroid cancer [54]. High abundances of CD8+ T lymphocytes also have been reported as a possible independent risk factor for recurrence prediction in differentiated thyroid cancer [55]. However, the role of immune cells in PTC is still unclear. Our preliminary observation could provide a perspective to explore the problem, further research is needed in the future.

However, there were some limitations to the present study, which should be considered when interpreting our results. First, transcriptomics analysis only could reflect some aspects of immune status rather than the global alterations. Second, The lacking of validation with another independent cohort is also a limitation of the study. Third, the reliability of our molecular results was still challenged by lacking in vitro or in vivo experiments.

As we look to the future, many questions remain. For example, relationships between immunogenomics, proteomics, and metabolomics should be explored to further delineate global immunological changes in PTC. Of importance, the potential relationship between disturbed immunogenomes and premalignant lesions should be further explored. We anticipate that this prognostic signature may be of great clinical import. We systematically analyzed the role of IRGs in the monitoring of the initiation and prognosis of PTC. Our findings have provided novel insights that could yield new immunotherapies in PTC.

## MATERIALS AND METHODS

### Clinical samples and data acquisition

Transcriptome RNA-sequencing data of PTC samples were downloaded from the TCGA data portal (https://cancergenome.nih.gov/), which contained data from 493 primary PTC and 58 non-tumor tissues. Raw count data was downloaded for further analyses. Clinical information for these patients was downloaded and extracted. We also derived a list of IRGs via the Immunology Database and Analysis Portal (ImmPort) database [56]. ImmPort is a database that updates immunology data accurately and timely. Data shared through ImmPort is a powerful foundation of immunology research. More importantly, the database provides a list of IRGs for cancer researches. These genes were identified to actively participate in the process of immune activity.

### Differential gene analysis

To selected IRGs involved in the onset of PTC, differentially expressed IRGs between PTC and adjacent non-tumor thyroid samples were screened via the R software edgeR package (http://bioconductor.org/packages/edgeR/) [57]. Trimmed mean of M values (TMM) implemented in the edgeR Bioconductor package was used to normalize the raw data. We performed differential gene analysis of all transcriptional data, setting a false discovery rate (FDR) < 0.05 and a log2 |fold change| > 1 as the cutoff values. Differentially expressed IRGs were then extracted from all differentially expressed genes. Functional enrichment analyses, via the GO and KEGG pathways [58–62], were conducted to explore potential molecular mechanisms of the differentially expressed IRGs.

### Survival analysis

Considering the generally favorable prognoses of most cases of PTC, the number of death events is small related to overall survival. We chose PRI as the primary endpoint, and all follow-up data was derived from TCGA's Pan-Cancer Atlas [63]. A log2 (normalized value + 1) data format was used for survival analysis. Survival-associated IRGs were selected by univariate COX analysis, which was conducted using the R software survival package. IRGs which were significantly related to PFI survival were also submitted for functional enrichment analysis.

### Molecular characteristics of hub IRGs

Differentially expressed IRGs which were significantly correlated to clinical outcomes of PTC patients were identified as hub IRGs. As these IRGs may have clinical applications, their clinical values were also systematically explored. Copy number alterations data was obtained from Cbioportal (http://www.cbioportal.org/) [64,65]. To explore the interactions between these genes, the PPI network was constructed based on data gleaned from the STRING online database (https://string-db.org/). PPI network could display many interactions that connect with hub genes directly or indirectly. The PPI result was displayed using Cytoscape software version 3.6.1 66. We also focused on their regulatory mechanisms. TFs are important molecules that directly control the degree of gene expression. Hence, it is necessary to explore how TFs that have potential ability in regulating these clinically relevant IRGs. Cistrome Cancer is a data source that integrates cancer genomics data from TCGA with over twenty-three thousands of ChIP-seq and chromatin accessibility profiles to provide the regulatory links between TFs and transcriptomes. The Cistrome Cancer database is a valuable resource for experimental and computational cancer biology research and contains a total of 318 TFs and. 67. We extracted clinically relevant TFs to construct the regulatory network of the current IRGs and potential TFs.

### Development of the immune-related gene-based prognostic index (IRGPI)

Hub IRGs were submitted for multivariate analyses, with integrated IRGs remaining as independent prognostic indicators to develop the IRGPI. The IRGPI was constructed based on expression data multiplied by the Cox regression coefficient. Patients were divided into high- and low-risk groups about the median PI value. The prognostic value of the PI was assessed in patients with different subtypes of PTC. The TIMER online database analyzes and visualizes the abundances of tumor-infiltrating immune cells [68]. TIMER reanalyzes gene expression data, which includes 10,897 samples across 32 cancer types from TCGA to estimate the abundance of six subtypes of tumor-infiltrating immune cells, including B cells, CD4 T cells, CD8 T cells, macrophages, neutrophils, and dendritic cells. Hence, it can be easily used for determining the relationship between immune cells infiltration and other parameters. We downloaded immune infiltrate levels of PTC patients and calculated associations between the IRGPI and immune cells infiltration.

### Statistical analysis

Gene functional enrichment analyses were conducted based on the R software clusterProfiler package of for identifying biological themes among gene clusters [69]. The AUC of the survival ROC curve was calculated via the survivalROC R software package to validate the performance of the prognostic signature [70]. Differences among clinical parameters were tested using independent *t*-tests. *P*-values of less than 0.05 were considered statistically significant.
